# Infant Diet

**Published:** 1891-12

**Authors:** 


### INFANT DIET.

One of the most frequent sources of danger to infants is the sudden weaning on fresh cow's milk and water. The massive curds formed from cow's milk are frequently beyond the feeble digestive powers of the infant. Dilution will partially remedy the matter, but cannot as a rule be carried to a sufficient extent without unduly increasing the bulk of the food. Boiled milk not only is to a certain degree sterilized, but clots less firmly than raw milk.

Another grave error is the prolonged use of artificially digested foods. The power of digesting casein becomes gradually less, nutrition is impaired, and the child tends to become flabby, anæmic, soft in bone.

Still another error consists in the furnishing of an insufficient amount of nutritive material. The capacity of the stomach being limited, it may be impossible for it to take a sufficient quantity of a digestably diluted mixture to supply the material (as casein) required for growth and nutrition. The difficulty may be overcome by the addition of cream or some good preparation of meat juice (bovinine). Food deficient in fat, as well as that deficient in protein, is certain to cause trouble. Those deprived of these two elements are often large and fat, but flabby, anæmic, and rachitic; yet most of the artificial foods upon which children are so largely fed are almost wholly lacking in fat and to a less degree in proteids.

The presence of an antiscorbutic element in the food is also a matter of prime importance. Fresh milk contains the element in sufficient quantity, but all farinaceous foods, and all the dry artificial foods, are decidedly deficient in this regard.—DR. CHEADLE.

